# Fire Behavior and Failure Model of Multilayered Wood Flour/HDPE/Polycarbonate Composites with a Sandwich Structure

**DOI:** 10.3390/polym14142833

**Published:** 2022-07-12

**Authors:** Jingfa Zhang, Ahmed Koubaa, Dan Xing, Haigang Wang, Yubo Tao, Xiang-Ming Wang, Peng Li

**Affiliations:** 1 Forests Research Institute, Université du Québec en Abitibi-Témiscamingue, Rouyn-Noranda, QC J9X 5E4, Canada; jingfa.zhang@uqat.ca (J.Z.); dan.xing@uqat.ca (D.X.); 2 State Key Laboratory of Biobased Material and Green Papermaking, Qilu University of Technology, Shandong Academy of Sciences, Jinan 250353, China; taoyubo@qlu.edu.cn (Y.T.); lipeng@qlu.edu.cn (P.L.); 3 Key Laboratory of Bio-Based Materials Science and Technology (Ministry of Education), Northeast Forestry University, Harbin 150040, China; hgwang@nefu.edu.cn; 4 New Construction Materials Group, Québec, QC G1V 4C7, Canada; xiang-ming.wang@fpinnovations.ca

**Keywords:** polymer composites, sandwich structure, thermocompression, fire behavior, mechanical properties, finite element analysis (FEA)

## Abstract

The flame retardancy of wood–polymer composites significantly affects their potential applications. Thus, multilayered wood flour/high-density polyethylene (HDPE)/polycarbonate (PC) composites were prepared via thermocompression to improve the fire retardancy of wood–polymer composites in this paper. Thermal degradation behavior, flame retardancy, and flexural strengths of the resulting composites were investigated using a thermogravimetric analysis, cone calorimetry, and mechanical testing machine, respectively. Results revealed that the boric acid treatment reduced the heat release rate and total heat release of the wood flour/HDPE composites and increased their mass of residues. However, boric acid reduced the flexural strength of the resulting composites. The combustion test indicated that PC cap layers suppressed the combustion of the resulting composites via the formation of carbon layers. Adding PC layers reduced heat release and increased the flexural strength of the resulting composites. Finally, the failure mode of the multilayered wood flour/HDPE/PC composites in the three-point flexural test was simulated by finite element analysis.

## 1. Introduction

Wood–polymer composites (WPCs) consisting of wood fibers and polymers have become a successful commercial product because of their low cost, dimensional stability, water resistance, corrosion resistance, and environmental friendliness [1,2,3]. They have been widely used as building and automotive materials [4]. A previous report shows that WPC terrace boards account for 85% of this in Europe [5]. However, wood fibers and polymers are inherently ignitable, resulting in WPCs with a high fire risk [6]. The inflammability of WPCs limits their utilization in several applications, especially for construction. Hence, improving WPCs’ flame retardancy is important and necessary. There have been a large number of studies concerning the improvement of fire resistance in WPCs [7,8,9]. However, most of these studies improved the fire retardancy of composites by adding flame retardants.

Generally, flame retardants, such as halogenated retardants, magnesium hydroxide (MH), expandable graphite (EG), boric acid (BA), and ammonium polyphosphate (APP) were combined into WPCs to produce flame-retardant composites [10,11]. Halogenated retardants were gradually abandoned since they were harmful to human health [6]. Although these flame retardants improved WPCs’ fire retardancy [12,13,14,15], their efficiency and mechanisms differed. Stark et al. [16] found that the effects of magnesium hydroxide and ammonium polyphosphate on flame performance are better than that of zinc borate. EG has one of the highest potentials for flame retardancy in WPCs, since it shows the best performance compared to APP and nitrogen-containing flame retardants [17]. The flame retardancy of WPCs depends on the chemical structure of the flame retardant when the quantity used is adequate. Hence, hybrid flame retardants were combined into WPCs [18,19]. Furthermore, flame retardants were directly mixed with lignocellulosic materials and polymers to get a uniform distribution. To meet flame retardancy requirements, flame–retardants loading must be more than 20 percent for WPCs, decreasing WPCs’ mechanical properties [6].

Recently, an increasing number of functional multilayer WPCs have been fabricated by coextrusion or thermoconsolidation. Compared with homogeneous composites, multilayered WPCs are made of different materials with various natures and have many advanced characteristics, such as being water resistant, acting as oxygen barriers, and having high toughness and photoluminescence [20,21,22,23]. The cap layer plays an important role in the function of multilayered WPCs. A WPCs cap layer with EG can significantly reduce the heat and smoke release of multilayered WPCs [6]. However, not all flame retardants can be used in the sandwiched structure to achieve gradient flame retardancy. The incorporation of APP caused greater heat release and smoke production for WPC with a sandwich structure than for the single-layer WPC. Moreover, researchers have also explored the thermal degradation kinetics of multilayered WPC [24]. Basalt-fiber-reinforced high-density polyethylene (HDPE) shells reduce the flammability of co-extruded wood–polymer composites [25]. Carbon fiber and glass–fiber–reinforced composites have also been used as shells to prepare lightweight structural materials with good fire retardancy [26,27].

Hence, the properties of multilayered composites can be controlled by adding a different functional cap layer. Polycarbonate (PC), a kind of engineering plastic, has been widely used in the electrical, construction, transportation, and automotive industries due to its advanced characteristics [28]. PC consists of aromatic rings and carbonates with a conjugate system, resulting in its dimensional stability, flame resistance, high heat distortion temperature, and outstanding mechanical properties [29,30,31]. Moreover, wood fiber and PC are hydrophilic due to their functional groups, such as hydroxyl and carboxyl. Hence, good compatibility between PCs and wood fibers is observed [32]. PC–based biocomposites have great potential for developing the WPC industry because of their good properties.

This study introduced polycarbonate as shell layers into sandwich wood–polymer composites to improve their fire retardancy and mechanical properties. The fire behaviors of the sandwich composites were explored, and the thermal degradation mechanisms of composites were investigated. The general objective of this study was to improve the fire retardancy of WPCs and their mechanical properties. The specific objectives were: (1) improve the thermal stability of the wood flour by using a boric acid treatment and then fill the wood into HDPE to improve the fire-retardancy of WPCs; (2) introduce PC into the composites to improve their fire-retardant and mechanical properties; (3) explore the failure mode of the multilayered wood flour/HDPE/polycarbonate composites with a sandwich structure. This study would promote using sandwich wood–polymer composites in construction, especially for non–structural applications.

## 2. Materials and Methods

### 2.1. Materials

Aspen veneer (*Populus tremuloides* Michx), supplied by LVLGLOBAL (Ville–Marie, Québec, QC, Canada) was ground using a hammer-mill grinder (RETSCH knife grinder) to obtain wood flour (40–80 mesh). Polycarbonate with a density of 1.2 g∙cm^−3^, commercial-grade Makrolon 6485, was purchased from Polyone Co. (Englewood, CO, USA). The melting flow index of the PC is 10 g∙10 min^−1^ (according to ISO 1133, 300 °C, 1.2 kg). The UL 94 flame retardancy leave of the pure PC was V-0 grade. Goodfellow Corp. (Henderson, NV, USA) provided the HDPE, a semicrystalline polymer (typically 70–80%) with a melt index of 9.0 g∙10 min^−1^ and a density of 0.95 g∙cm^−3^. Honeywell (Wuhan, China) supplied the Maleic modified polyethylene (MAPE, A-C^®^ 575P) with a density of 0.92 g∙cm^−3^. Sigma-Aldrich Canada (Oakville, ON, Canada) provided the analytical reagent-grade boric acid (BA) powder.

### 2.2. Preparation of Multilayered Wood Flour/HDPE/PC Composites

**Figure 1:**
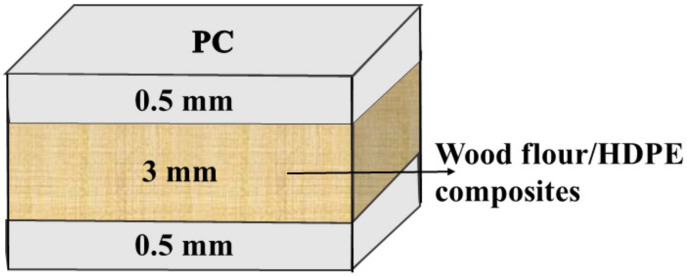
Schematic diagram of the multilayered WPCs with sandwich structure.

A boric acid aqueous solution of 5% modified the wood flour. Wood flour was immersed in the above boric acid solution for 2 h and then dried in a laboratory oven at 105 °C for 48 h. The final moisture content of the wood flour was lower than 3%. The wood flour and HDPE particles were blended using a counter-rotating twin-screw extruder at 180 °C with a speed of 50 rpm (HAAKE PolyLab OS Rheodrive 7, HAKKE, Karlsruhe, Germany) according to the formulations in Table 1. The mixtures were then compressed into samples of different sizes for testing (sample size is based on relative standards) using a hydraulic press (LabEcon300, Fontijne, Delft, The Netherlands) at 230 °C. The pressure and pressing time were 200 MPa and 5 min, respectively. The multilayered WPCs were fabricated by combining molten PC and wood-flour/HDPE sheets using the hot press. The pressing conditions were the same as those mentioned above. The thickness of the PC cap layer was 0.5 mm, and the schematic diagram of the multilayered WPCs is shown in Figure 1.

### 2.3. Characterizations of the Resulting Composites

The thermal degradation behavior of the resulting wood flour/HDPE/PC composites was analyzed by TG tests under a nitrogen atmosphere. The sample blocks of 10 mg were heated from 50 to 600 °C at a rate of 10 °C∙min^−1^ using a Q600 thermogravimetric (TG) analyzer (TA, New Castle, DE, USA). Three-point flexural tests were conducted according to ASTM D 790 with a span-to-depth ratio of 16:1. The crosshead speed was 2 mm∙min^−1^. Specimens measuring 127 mm × 13 mm × 4 mm were loaded using a Zwick universal mechanical test bench (Z020, Zwick Roell Group, Ulm, Germany). The combustion behavior of the resulting composites was investigated using a cone calorimeter (CONE) with an external heat flux of 50 kW∙m^−2^. The specimens, measuring 100 × 100 × 4 mm, were wrapped in aluminum paper and placed horizontally according to the ASTM E 1354-2009 standard. The strength values were obtained and averaged based on five measurements, while three replicates per composite formulation were carried out for the cone calorimeter test. Finite element analysis (FEA) of the three-point flexural test of the resulting multilayered composites was conducted using a FEA software. For simplicity, wood flour/HDPE composites should be isotropic materials. The specimens for FEA were the same dimensions as the flexural specimens according to ASTM D 790.

## 3. Results and Discussion

### 3.1. Thermal Degradation Behavior of the Resulting Composites

**Figure 2:**
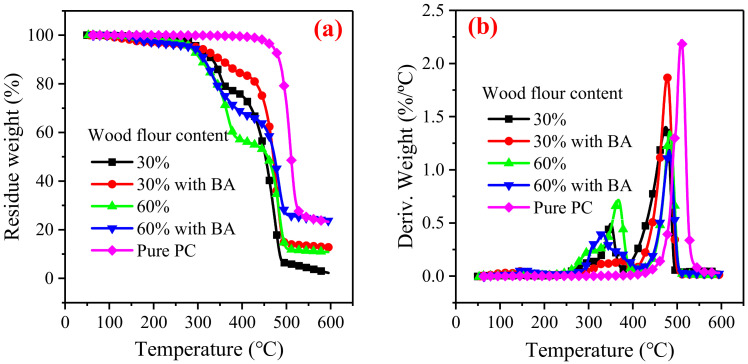
TG (**a**) and DTG (**b**) curves of the wood flour/HDPE composites and pure polycarbonate.

The thermal degradation of the wood flour/HDPE composites and pure PC were analyzed and compared (Figure 2). Char residue and thermal decomposition peak temperatures were higher for pure PC than for the wood flour/HDPE composites. This indicates that the thermal stability of PC is higher than that of the wood flour/HDPE composites, which can be explained by the fact that PC has a lot of aromatic rings which have higher stability than the fatty chains of HDPE. The thermogravimetric (DTG) curves show that the thermal degradation of the wood flour/HDPE composites was divided into two stages resulting from the wood flour degradation and the HDPE decomposition. The mass loss of the first decomposition stage increased with wood flour content and was attributed to wood degradation. The char residue of the wood flour/HDPE composites increased with an increase in wood flour content. The boric acid treatment reduced the degradation rate of the wood flour/HDPE composites and promoted the formation of char residues, consistent with previous findings [11,33]. Boric acid changed wood’s chemical structure, resulting in easy carbonization [34]. Furthermore, the borated biochar acts as thermal insulation, slowing down polymer matrix degradation.

### 3.2. Inflammability of the Resulting Composites

#### 3.2.1. Single-Layer Wood Flour/HDPE Composites with and without Boric Acid Treatment

**Figure 3:**
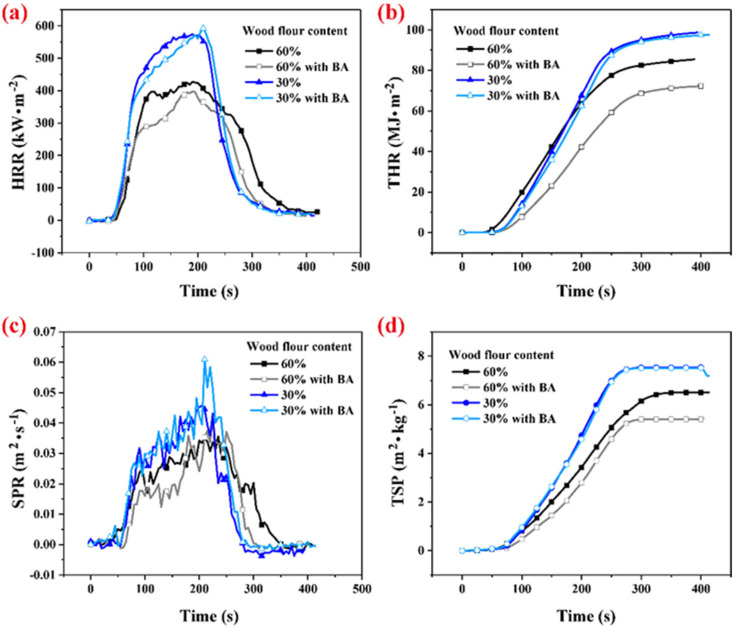
Flame–retardant properties of wood flour/HDPE composites with and without boric acid (BA). (**a**) Heat release rate (HRR), (**b**) total heat release THR, (**c**) smoke production rate (SPR), and (**d**) total smoke production (TSP).

Boric acid treatments reduced the WPCs’ heat release rates (HRR), especially for WPCs filled with high wood flour content (Figure 3a). The HRR values of WPCs with BA were lower than those without BA for testing times between 100 and 200 s. The effect of boric acid on the total heat release (THR) of WPCs with 30% wood flour content was almost negligible (Table 2). However, the THR value of WPCs with BA decreased by 15.6% compared to WPCs without BA when the wood flour content was 60% (Figure 3b). Previous studies have shown that the slope of THR curves is representative of flame spread [14]. Thus, the flame spread of WPCs decreased after adding BA. Boric acid had little impact on the smoke production rate (SPR) of the wood flour/HDPE composites (Figure 3c). The change in total smoke production (TSP) of WPCs was the same as that of THR after the boric acid treatment (Figure 3d).

The effect of boric acid on flame retardancy and other properties of wood materials has been reported in previous studies [33,35,36]. Boric acid reacts with wood and forms a new chemical structure, changing the thermal degradation chemical pathway of wood [34]. In the presence of boric acid, cellulose and hemicellulose are more easily thermally degraded to biochar rather than combustible gas. In other words, a boric acid treatment increased char residue during wood combustion. The formed carbon layer insulates oxygen and heat, which slows the combustion of the composite, suppressing its HRR. This can explain the decrease in HRR of the wood flour/HDPE composites with boric acid for testing between 100 and 200 s. The effect of boric acid on flame retardancy was more obvious for WPCs with high wood flour content than for WPCs with low wood flour content, indicating boric acid barely affects the polymer matrix. However, the char layer resulting from wood flour suppressed the HRR of the polymer resin in composites (Figure 3a).

#### 3.2.2. Single–Layer Borated Wood Flour/HDPE Composites

**Figure 4:**
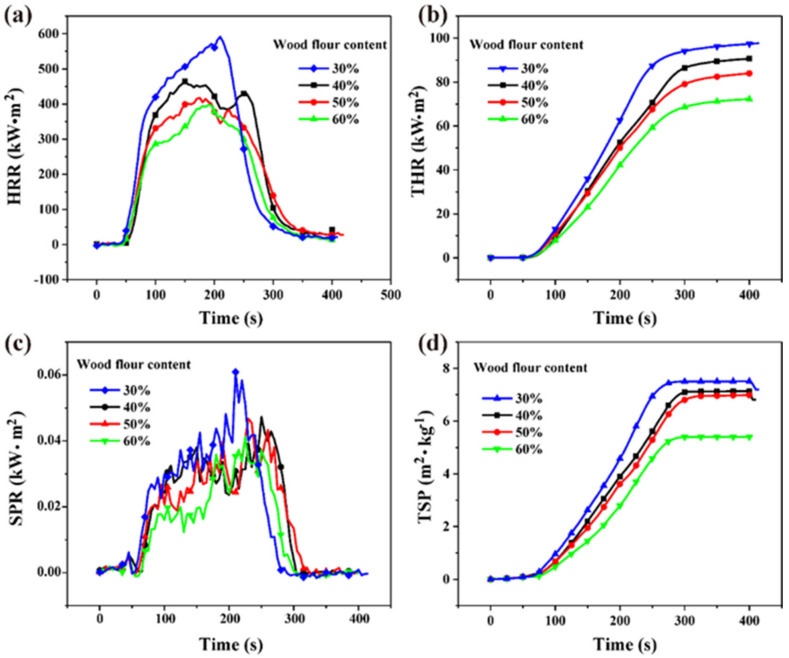
Combustion parameters of the borated wood flour/HDPE composites. (**a**) heat release rate (HRR), (**b**) total heat release THR, (**c**) smoke production rate (SPR), and (**d**) total smoke production (TSP).

The HRR decreased with increased wood flour content (Figure 4a). Similar trends were observed in THR, SPR, and TSP (Figure 4b–d). Furthermore, the combustion time lengthened with increasing wood flour content. These results indicate that borated wood flour positively impacts the fire retardancy of polymer composites, which is consistent with previous findings [12]. Wood flour contains a lot of oxygen and aromatic chemical groups, thus having a lower heat release than HDPE. These results, along with those in the literature, indicate that boric acid can improve the flame retardancy of wood flour and reduce the HRR, THR, SPR, and TSP of wood during combustion. Therefore, WPCs containing higher proportions of borated wood flour have good flame retardancy. On the other hand, char residues formed by the degradation of the wood flour increase with increasing wood flour content, thereby suppressing the combustion of the polymer matrix [9,12].

#### 3.2.3. Multilayered Wood Flour/HDPE/PC Composites

**Figure 5:**
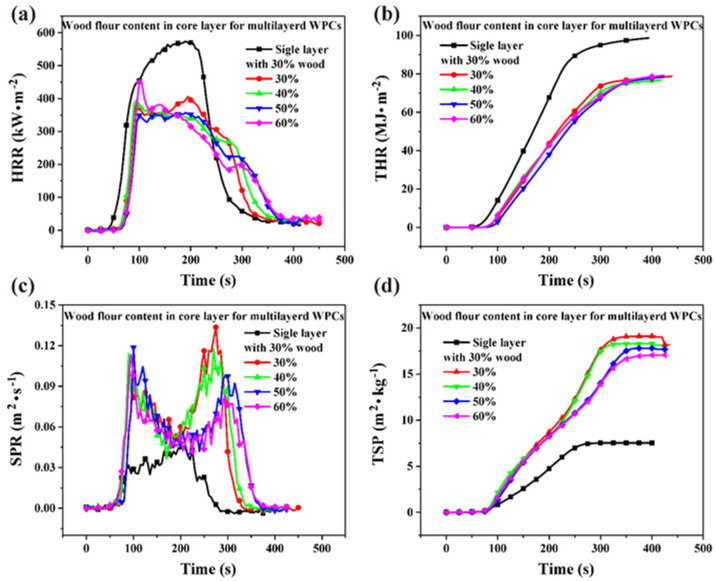
Combustion parameters of the multilayered wood flour/HDPE/PC composites. (**a**) heat release rate (HRR), (**b**) total heat release THR, (**c**) smoke production rate (SPR), and (**d**) total smoke production (TSP).

Compared to single-layer WPCs, both HRR and THR of multilayered wood flour/HDPE/PC composites were lower, and the combustion time was longer (Figure 5a,b). The wood flour content in the core layer had no obvious effect on the combustion of the multilayered composites. THR decreased by 20.2% from 98.7 MJ∙m^−2^ for the single wood flour/HDPE composites to 78.8 MJ∙m^−2^ for the multilayered wood flour/HDPE/PC composites. All observations show that PC sheets acting as cap and base layers have great fire-retarding effects for WPCs. However, SPR and TSP increased after covering the composites with PC sheets (Figure 5c,d). The SPR curves of multilayered wood flour/HDPE/PC composites showed two obvious peaks ascribed to the PC cap layers on the top and bottom of the composites. Furthermore, the TSP curves of the multilayered composites could be divided into two steps, which is in good agreement with SPR curves.

**Figure 6:**
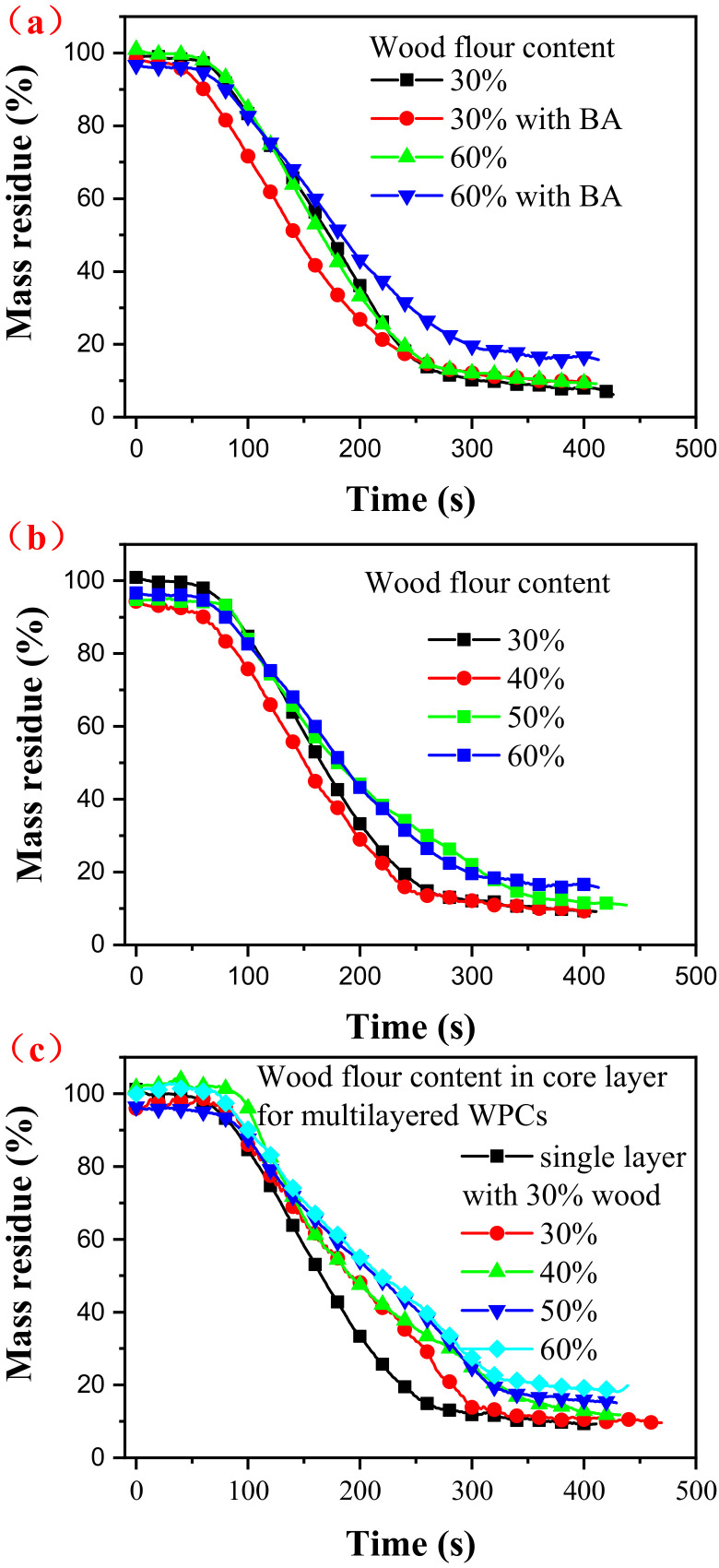
Mass residue curves of wood flour/HDPE composites (**a**), boric acid treated wood flour/HDPE composites (**b**), and multilayered wood flour/HDPE/PC composites (**c**).

As shown in Figure 4, the mass residue of the wood flour/HDPE composites increased after boric acid treatment (Figure 6a). This can be explained by the boric acid promoting the char formation from wood flour in composites [34]. Furthermore, the mass residue increased with increasing wood flour content. A similar phenomenon was observed for the multilayered wood flour/HDPE/PC composites (Figure 6c). Different from the single-layer wood flour/HDPE composites, the mass residue curves of the multilayered composites can be divided into stages because of their sandwich structure. Furthermore, time to ignition (TTI) was longer for multilayered wood flour/HDPE/PC composites than for the single-layer wood flour/HDPE composites (Table 2). PC has good thermal stability due to its aromatic ring structure and low thermal conductivity.

**Figure 7:**
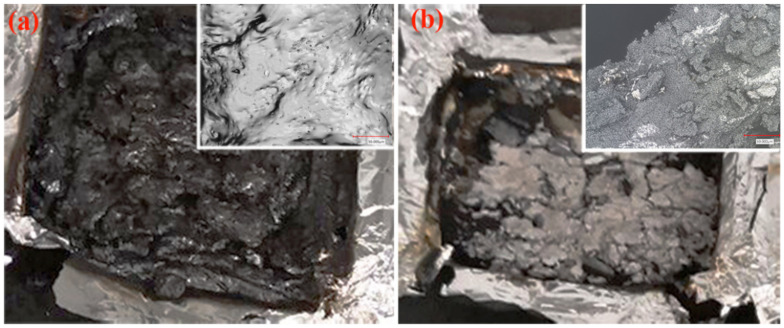
Digital photos and micro-morphology of char formation on the resulting composites, (**a**) multilayered wood flour/HDPE/PC composites, and (**b**) single-layer wood flour/HDPE composites.

The above results indicated that the PC cap layer improved the flame retardancy of the resulting composites, which can be attributed to the aromatic ring of PC chains. The PC used in this study is self-extinguishing and showed a V-0 grade in the UL94 test. The multilayered composites featured a continuous and dense carbon layer, which can be attributed to the combustion of the PC cap layers (Figure 7a). In contrast, the char formed from wood flour/HDPE composites was less fluffy (Figure 7b).

**Figure 8:**
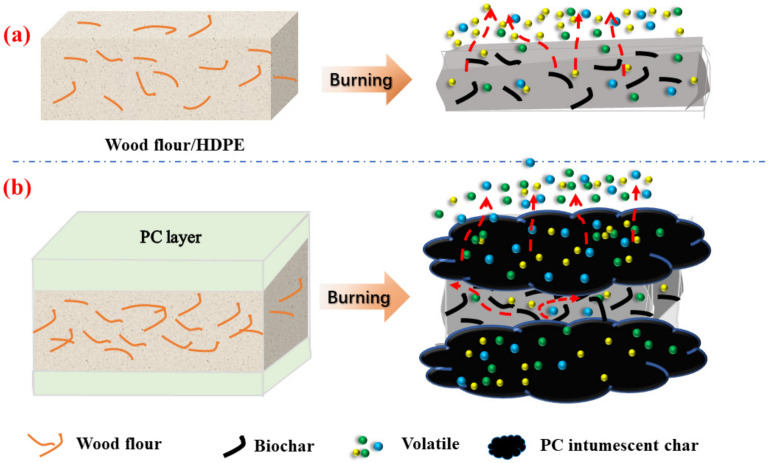
The schematic of smoke and flame suppression mechanism of single-layer (**a**) and multilayered WPCs (**b**).

Figure 8 shows the schematic of combustion mechanisms of the single wood flour/HDPE composites and the multilayered composites. Wood flour and HDPE decomposed and produced flammable volatiles when burning. For boric acid-treated wood flour/HDPE composites, wood flour was catalyzed into biochar by boric acid when burning, resulting in increased mass residue. In this case, HDPE mainly produced flammable volatiles (Figure 8a). The PC cap layer suppressed the volatiles’ effluence and isolated them from the oxygen. The flaming of the resulting composites was suppressed, resulting in high char residue. Moreover, the PC layer protected the base layer from decomposition by reducing heat transfer [9]. In summary, the PC cap layer acted as a protective layer, improving the flame retardancy of biocomposites.

### 3.3. Mechanical Properties

**Figure 9:**
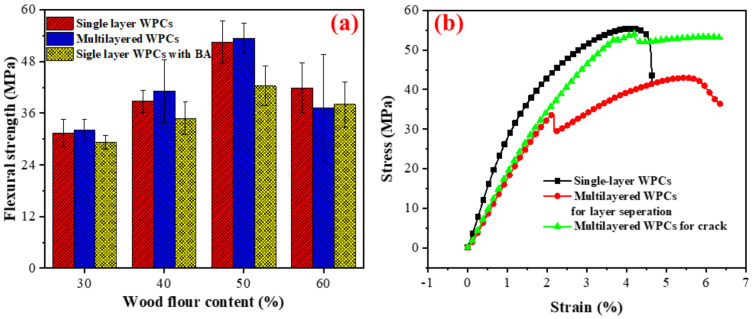
(**a**) Flexural strength of the resulting composites and (**b**) the strain-stress plot of the resulting composites with 50% wood flour content.

For both single-layer and multilayered WPCs, the flexural strength of the resulting composites increased with increasing wood flour content when the wood flour content was lower than 50% (Figure 9a). The flexural strength subsequently decreased with adding more wood flour. This can be explained by the threshold theory proposed in the previous study [37]. The increase in the flexural strength is ascribed to the effect of wood flour reinforcement when MAPE was used as a coupling agent [38]. However, the wood flour aggregation induces stress concentration when its content exceeds threshold values, leading to composite failure. The boric acid treatment reduced the flexural strength of the single-layer WPCs, which agrees with previous findings [11]. Boric acid treatment changes wood flour’s molecular structure and crystallization, decreasing its mechanical strength. In contrast, compared with single-layer wood flour/HDPE composites, the flexural strength showed an increasing trend for multilayered wood flour/HDPE/PC composites. This increase can be attributed to the reinforcement of the PC sheet. High-performance shell materials increase the strength of multilayered WPCs [25]. However, improving their interfacial adhesion is crucial to obtaining a high-mechanical-performance multilayered WPC.

The failure mode of single-layer wood flour/HDPE composites was different from that of the multilayered wood flour/HDPE/PC composites. Single-layer wood flour/HDPE composites broke into two parts directly after damage. The strain-stress curves showed that the stress of the single-layer composites dropped suddenly (Figure 9b). The failure of the multilayered WPCs resulted from damage to the wood flour/HDPE core layer or the separation of different layers. The stiffness of the wood flour/HDPE core layer is higher than that of the PC cap layer, but the strength is just the opposite. Therefore, crack initiation occurs to a greater extent in the core layer, leading to the failure of the resulting composites. Furthermore, debonding at the interface between the core layer and the shell layer will directly lead to the failure of the composites. The strain–stress curves of the multilayered composites represent the above two failure modes (Figure 9b). It can be seen that the debonding of the PC cap layer and core wood flour/HDPE layer suddenly reduced the stress. However, stress still increased subsequently with the strain increase. As to the damage to the wood flour/HDPE core layer, the stress decreased and stayed at a certain level due to the PC cap layer.

**Figure 10:**
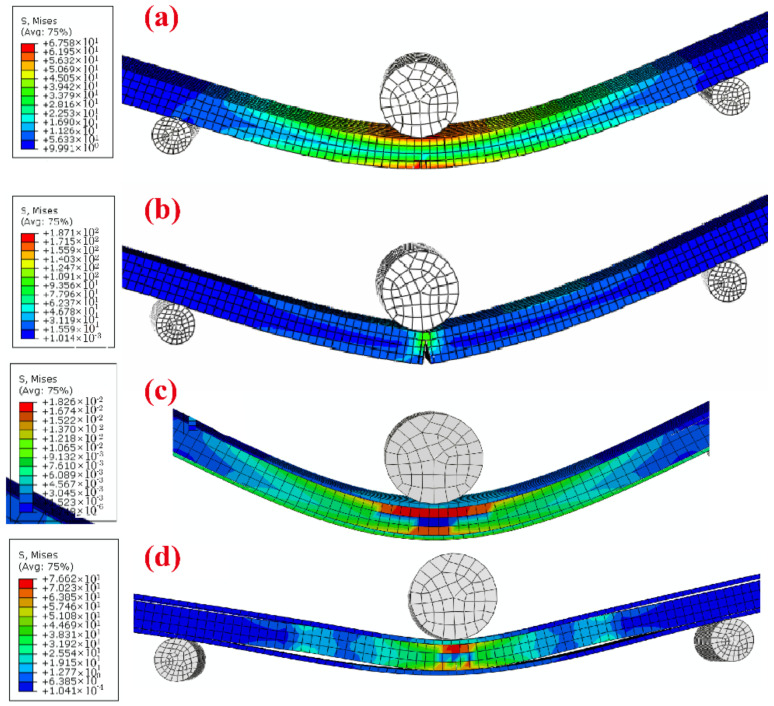
Schematic graph of the flexural samples for finite element modeling. Single-layer wood flour/HDPE composites (**a**,**b**), multilayered wood flour/HDPE/PC composites (**c**,**d**).

Additionally, FEA analyzed the failure mechanisms of the resulting composites. As shown in Figure 10, the stress distribution of the multilayered wood flour/HDPE/PC composites in the flexural test was simulated and analyzed using FEA under a constant load. The wood flour/HDPE composites were assumed to be homogeneous. It is well known that the lower surface of the sample will bear the greatest stress in the flexural test, as shown in Figure 10a. For single-layer wood/HDPE composites, cracks were initiated and expanded with increasing external load (Figure 10b). The simulated results of the multilayered composites showed that the greatest stress occurred in the WPC core layer instead of in the lower cap PC layer (Figure 10c). This observation can be explained by the fact that the modulus of elasticity of the wood flour/HDPE composite is larger than that of the pure PC, while the Poisson ratio is the opposite. Even so, the PC cap layer bore some stress when the external force was loaded, increasing the flexural strength of the resulting composites. The reinforcement effect of PC layers was lost when the interfacial bonding between the core layer and the cap layer failed with an increase in load and deflection values, as shown in Figure 10d. These observations explain why the flexural strength of multilayered wood flour/HDPE/PC composites was much lower than that of pure PC. In summary, to produce a high-performance multilayered WPC, the modulus and strength of the cap layer should be higher than those of the WPC core layer. In contrast, good compatibility between WPCs and cap layer materials is achieved.

## 4. Conclusions

Multilayered wood flour/HDPE/PC composites with a sandwich structure were prepared using a hot press at a relatively high temperature, and the effect of the PC cap layer on fire retardancy and mechanical strength of the resulting composites was explored. The PC layer formed a protective char layer, suppressing the combustion of the wood flour/HDPE core layer. Both boric acid treatment and the PC cap layer promoted char residue formation for the multilayered composites. This indicated both had positive effects on the flame retardancy of WPCs. The addition of the PC cap layer increased the flexural strength of the resulting composites, but the opposite happened with boric acid. High wood flour content in the wood flour/HDPE core layer showed beneficial effects on the flame retardancy and mechanical strength of the resulting multilayered composites. Furthermore, the failure mode of the multilayered wood flour/HDPE/PC composite in the three-point flexural test was simulated by finite element analysis. The result indicated that the elastic modulus of the cap layer materials and the interfacial bonding of different layers played important roles in the mechanical strength of the resulting composites.

## Figures and Tables

**Table 1:** Formulations of the wood flour/HDPE composites.

Samples	Wood Flour (wt.%)	HDPE (wt.%)	MAPE (wt.%)
WPC30	30	66	4
WPC40	40	56	4
WPC50	50	46	4
WPC60	60	36	4

**Table 2:** Cone calorimeter test data of the resulting composites with 30% wood flour content.

Samples	TTI (s)	THR (MJ∙m^−2^)	PHRR (kW∙m^−2^)	TSP (m^2^∙kg^−1^)
Wood flour/HDPE composites	48 (±5.0)	98.7 (±0.2)	578.1 (±7.9)	7.1 (±0.8)
Borated wood flour/HDPE composites	46 (±2.0)	97.7 (±1.1)	562.7 (±27.8)	7.2 (±0.3)
Multilayered WPCs	63.7 (±1.2)	78.8 (±1.2)	395.5 (±18.7)	19.2 (±0.85)

## Data Availability

Available data can be obtained from the corresponding author upon request.
